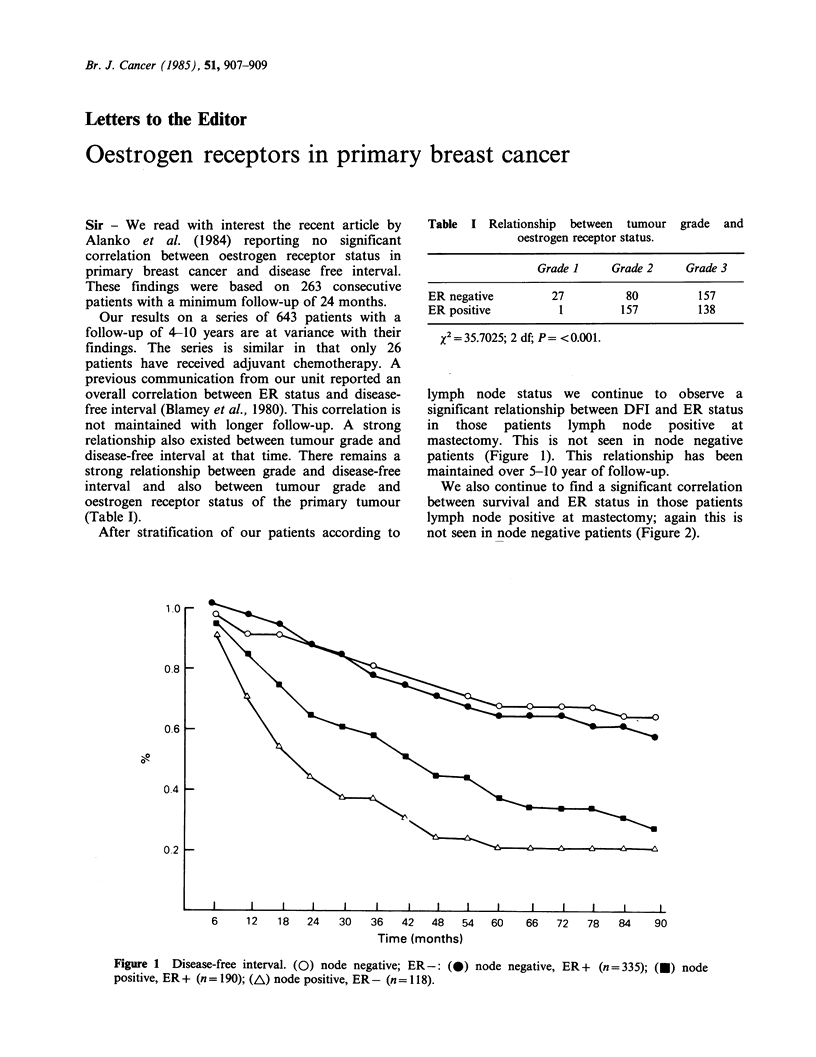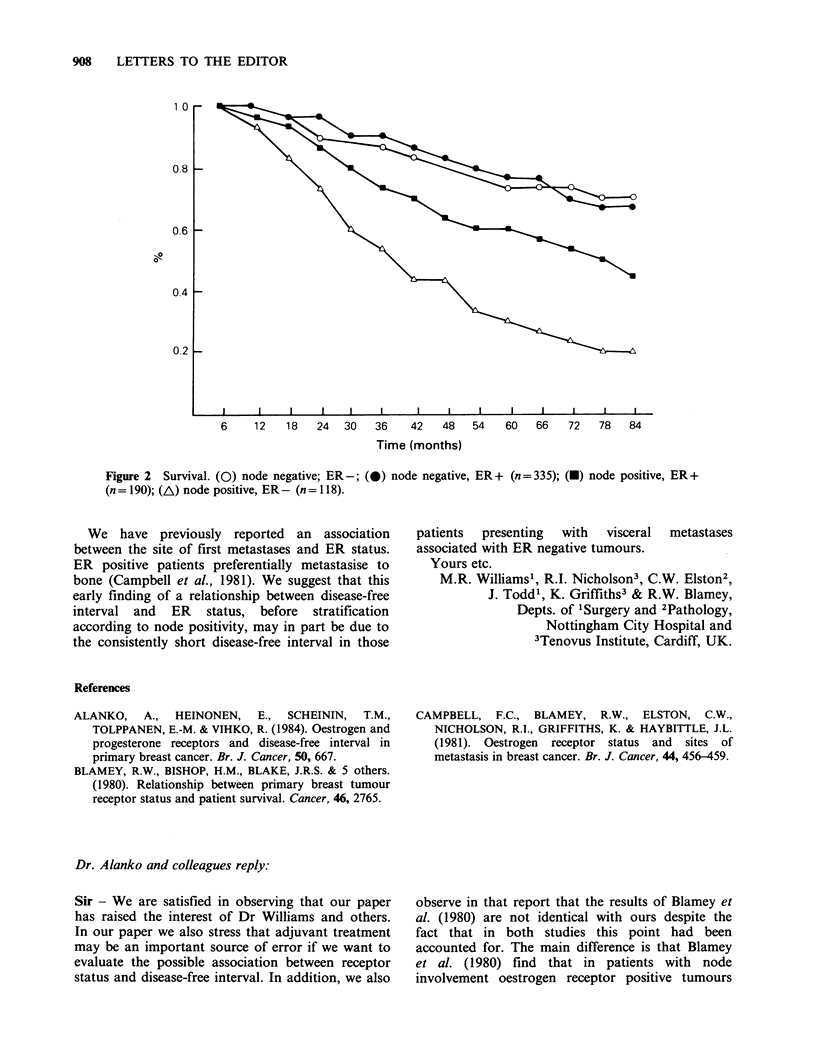# Oestrogen receptors in primary breast cancer.

**DOI:** 10.1038/bjc.1985.138

**Published:** 1985-06

**Authors:** M. R. Williams, R. I. Nicholson, C. W. Elston, J. Todd, K. Griffiths, R. W. Blamey


*Br. J. Cancer (1985)*, **51**, 907–909

### Letters to the Editor

### Oestrogen receptors in primary breast cancer

**Sir** – We read with interest the recent article by Alanko *et al.* (1984) reporting no significant correlation between oestrogen receptor status in primary breast cancer and disease free interval. These findings were based on 263 consecutive patients with a minimum follow-up of 24 months.

Our results on a series of 643 patients with a follow-up of 4–10 years are at variance with their findings. The series is similar in that only 26 patients have received adjuvant chemotherapy. A previous communication from our unit reported an overall correlation between ER status and disease-free interval (Blamey *et al.*, 1980). This correlation is not maintained with longer follow-up. A strong relationship also existed between tumour grade and disease-free interval at that time. There remains a strong relationship between grade and disease-free interval and also between tumour grade and oestrogen receptor status of the primary tumour (Table I).

After stratification of our patients according to lymph node status we continue to observe a significant relationship between DFI and ER status in those patients lymph node positive at mastectomy. This is not seen in node negative patients (Figure 1). This relationship has been maintained over 5–10 year of follow-up.

We also continue to find a significant correlation between survival and ER status in those patients lymph node positive at mastectomy; again this is not seen in node negative patients (Figure 2).

**Table I** Relationship between tumour grade and oestrogen receptor status.

| | *Grade 1* | *Grade 2* | *Grade 3* |
|---|---|---|---|
| ER negative | 27 | 80 | 157 |
| ER positive | 1 | 157 | 138 |

$\chi^2 = 35.7025$; 2 df; $P = < 0.001$.

**Figure 1** Disease-free interval. (○) node negative; ER−: (●) node negative, ER+ ($n$=335); (■) node positive, ER+ ($n$=190); (△) node positive, ER− ($n$=118).

**Figure 2** Survival. (○) node negative; ER−; (●) node negative, ER+ ($n=335$); (■) node positive, ER+ ($n=190$); (△) node positive, ER− ($n=118$).

We have previously reported an association between the site of first metastases and ER status. ER positive patients preferentially metastasise to bone (Campbell *et al.*, 1981). We suggest that this early finding of a relationship between disease-free interval and ER status, before stratification according to node positivity, may in part be due to the consistently short disease-free interval in those patients presenting with visceral metastases associated with ER negative tumours.

Yours etc.

M.R. Williams[1], R.I. Nicholson[3], C.W. Elston[2], J. Todd[1], K. Griffiths[3] & R.W. Blamey,
Depts. of [1]Surgery and [2]Pathology,
Nottingham City Hospital and
[3]Tenovus Institute, Cardiff, UK.

**References**

ALANKO, A., HEINONEN, E., SCHEININ, T.M., TOLPPANEN, E.-M. & VIHKO, R. (1984). Oestrogen and progesterone receptors and disease-free interval in primary breast cancer. *Br. J. Cancer*, **50**, 667.

BLAMEY, R.W., BISHOP, H.M., BLAKE, J.R.S. & 5 others. (1980). Relationship between primary breast tumour receptor status and patient survival. *Cancer*, **46**, 2765.

CAMPBELL, F.C., BLAMEY, R.W., ELSTON, C.W., NICHOLSON, R.I., GRIFFITHS, K. & HAYBITTLE, J.L. (1981). Oestrogen receptor status and sites of metastasis in breast cancer. *Br. J. Cancer*, **44**, 456–459.